# Fractionation methods and post-heat treatment shape in vitro protein and starch digestibility of oat protein ingredients

**DOI:** 10.1016/j.crfs.2026.101477

**Published:** 2026-06-15

**Authors:** Danny Tagle-Freire, Jesse J. Kuiper, Michiel Ohms, Yuanfu Chiang, Markus Stieger, Ciarán G. Forde, Maarten A.I. Schutyser

**Affiliations:** a Laboratory of Food Process Engineering, Wageningen University & Research, Bornse Weilanden 9, Wageningen, 6708 WG, the Netherlands; b Sensory Science and Eating Behaviour, Division of Human Nutrition and Health, Wageningen University & Research, Stippeneng 4, Wageningen, 6703 HD, the Netherlands

**Keywords:** Oat protein, Oat starch, Heat treatment, Wet extraction, Dry fractionation, INFOGEST

## Abstract

The digestibility of plant protein ingredients is influenced by processing, yet the roles of fractionation and post-heat treatment in oat ingredients remain poorly understood. This study investigated how dry fractionation, alkaline extraction–isoelectric precipitation (AE-IP), and water-only extraction influence composition, particle morphology, solubility, and in vitro protein and starch digestibility of oat protein ingredients. Oat protein concentrate produced by dry fractionation (OPC), oat protein isolate produced by AE-IP (OPI), and water-only extracted oat protein isolate (OPIW) showed distinct compositional and morphological characteristics. Protein digestibility was lowest for OPIW (26.3%), followed by OPI (45.3%), and highest for oat flour (66.2%) and OPC (61.4%). Despite similar protein profiles and evidence that proteins were not fully denatured prior to heating, OPIW exhibited low digestibility together with low solubility and sponge-like particle morphology. In contrast, starch digestibility showed limited variation among ingredients. Heating hydrated samples at 70 °C for 5 min induced starch gelatinization but did not affect protein digestibility, whereas heating at 110 °C for 5 min reduced protein digestibility by approximately 30% in flour and 21% in OPC, consistent with the loss of protein denaturation transitions measured by DSC. The results suggest that differences in digestibility among oat protein ingredients cannot be explained by composition alone and are associated with processing-induced changes during production. The findings further indicate that thermal effects on protein digestibility depend on the fractionation method, which may be relevant for selecting oat ingredients for thermally processed foods. Understanding how these processing-dependent differences translate to digestion kinetics and postprandial responses is an important next step.

## Highlights

• Fractionation method influenced oat ingredient morphology and solubility.
• Protein digestibility could not be explained by composition alone.
• Water-only extraction produced a poorly soluble ingredient with low digestibility.
• Heating at 110 °C reduced protein hydrolysis in flour and air-classified ingredient.
• Starch hydrolysis varied little among ingredients and was unaffected by heating.

## Graphical abstract

**Figure:**
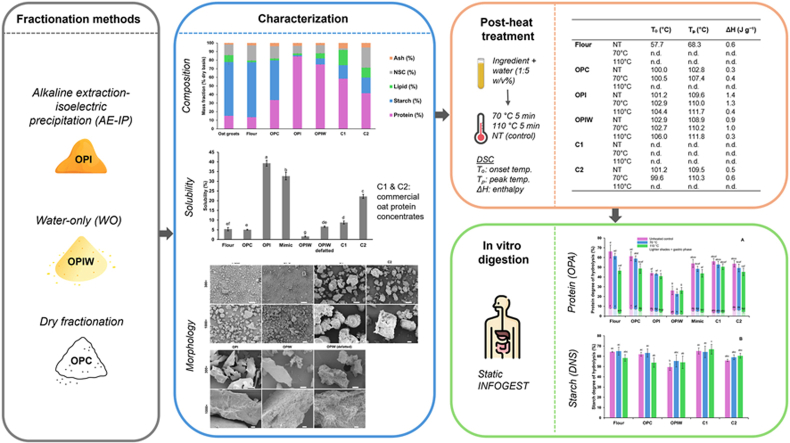

## 1 Introduction

The growing demand for sustainable, plant-based proteins has increased interest in cereals as alternative protein sources, particularly oats (*Avena sativa* L.). Oats combine relatively high protein content with a favorable amino acid composition and health-promoting components such as β-glucans (El Khoury et al., 2012; Poutanen et al., 2022). Oat proteins exhibit a Digestible Indispensable Amino Acid Score (DIAAS) of approximately 77, exceeding that of wheat and rice, and are increasingly explored for food applications beyond traditional cereal products, such as plant-based meat analogues and dairy alternatives (Li et al., 2025; Poutanen et al., 2022).

To obtain protein-enriched oat ingredients, fractionation is required. Conventional alkaline extraction followed by isoelectric precipitation (AE-IP) yields high-purity protein isolates but involves extensive water use, chemical inputs, and energy-intensive drying (Schutyser et al., 2025). More sustainable alternatives have therefore been developed, including dry fractionation by air classification and wet extraction using water only as the extraction medium, without pH adjustment (Lie-Piang et al., 2023). Beyond their environmental footprint, these routes generate ingredients with different protein–starch matrices, as differences in processing conditions influence separation efficiency and thereby the residual starch content in the resulting ingredients.

Because fractionation methods produce ingredients that differ in composition and structure, they may also influence how oat protein ingredients are digested. Oat protein ingredients often contain both protein and starch within the same matrix, particularly when produced by dry fractionation or water-only extraction (Lie-Piang et al., 2023). Thermal processing is particularly relevant in this context because oat protein ingredients are typically processed into whole foods, such as baked products or structured plant-based foods (Li et al., 2025). Heating can simultaneously modify both macronutrients within the ingredient matrix. Oat starch gelatinizes at approximately 60–65 °C, increasing enzyme accessibility and susceptibility to amylolysis, whereas oat globulins denature and aggregate at around 110 °C, which may reduce proteolysis through decreased solubility and enzymatic accessibility (Del Rio et al., 2022; Kaur et al., 2022; McLauchlan et al., 2024; Yang et al., 2023, 2024). The combined effects of fractionation method and subsequent heat load may therefore determine the digestibility of oat protein ingredients.

Despite growing interest in oat protein ingredients, research has largely focused on extraction efficiency, composition, and techno-functional properties, while comparatively little attention has been given to digestion in relation to fractionation methods and post-processing conditions of oats (Holopainen-Mantila et al., 2024; Li et al., 2025; Manzanilla-Valdez et al., 2024). Moreover, starch and protein digestibility are typically studied separately, even though both macronutrients coexist within the same ingredient matrix and are affected by processing-induced changes such as starch gelatinization and protein denaturation. Evaluating both may therefore provide a more comprehensive assessment of the effects of processing on oat protein ingredients.

The objective of this study was to investigate how fractionation methods and post-heat treatment influence the in vitro protein and starch digestibility of oat protein ingredients. Protein-enriched oat ingredients produced by dry fractionation, AE-IP, and water-only extraction were characterized in terms of composition, protein profile, morphology, solubility, color, and thermal transitions (protein denaturation and starch gelatinization). Ingredients were either left untreated or subjected to post-heat treatments at 70 °C or 110 °C for 5 min. Thermal transitions were subsequently evaluated to assess heat-induced changes in starch gelatinization and protein denaturation among ingredients. Finally, in vitro protein and starch digestibility were assessed using the standardized static INFOGEST in vitro digestion protocol (Brodkorb et al., 2019) to determine how fractionation method and post-heat treatment influence digestibility.

## 2 Materials and methods

### 2.1 Materials

Dehulled oat groats (Harivenesa; Navarra, Spain) supplied by Danone Research & Innovation (Utrecht, The Netherlands) were obtained from a single batch and used as the raw material. According to the supplier, the groats underwent kilning consisting of steaming (100.5 °C for 25 min), radiator heating (80 °C for 10 min, 70 °C for 10 min, 65 °C for 10 min, and 60 °C for 10 min), and cooling from 60 to 20 °C over 15 min. Two commercial oat protein concentrates were included as benchmark ingredients and provided by Fazer Mills (Lahti, Finland) and Lantmännen Biorefineries (Norrköping, Sweden). These ingredients were randomly coded as commercial oat protein concentrate 1 (C1) and commercial oat protein concentrate 2 (C2).

Unless otherwise stated, all chemicals and reagents were purchased from Sigma-Aldrich (St. Louis, MO, USA), including pepsin from porcine gastric mucosa (≥3200 U mg^−1^ protein; Cat. No. P6887) and pancreatin from porcine pancreas (8× USP specifications; Cat. No. P7545). The same enzyme batches were used throughout the study.

### 2.2 Fractionation methods

**Fig. 1:**
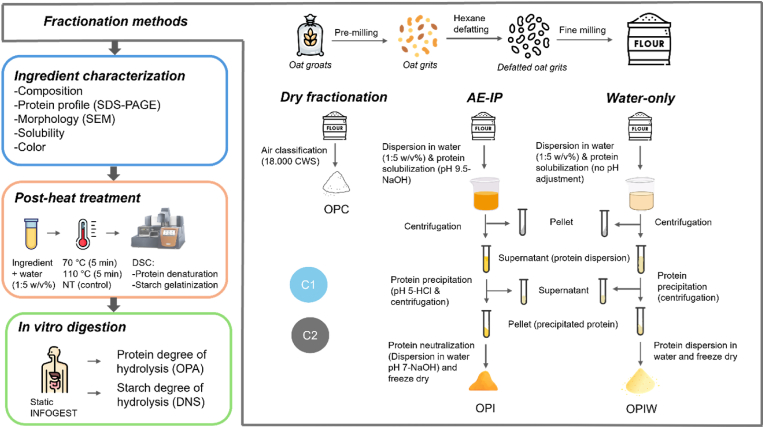
Methodology overview of the study. SEM, scanning electron microscopy; SDS-PAGE, sodium dodecyl sulfate–polyacrylamide gel electrophoresis; DSC, differential scanning calorimetry; OPA, o-phthaldialdehyde assay; DNS, dinitrosalicylic acid assay; CWS, classifier wheel speed; OPC, oat protein concentrate from dry fractionation; AE-IP, alkaline extraction–isoelectric precipitation; OPI, oat protein isolate from AE-IP; OPIW, oat protein isolate from the water-only process; C1 and C2, commercial oat protein concentrates. Icons were taken and adapted from BioRender.com and Flaticon.com.

Three fractionation methods were evaluated in this study to obtain oat protein ingredients: dry fractionation by air classification, conventional wet alkaline extraction followed by isoelectric precipitation (AE-IP), and water-only extraction (Fig. 1). Defatted oat flour served as the starting material for all processes. To produce defatted oat flour, dehulled oat groats were pre-milled using a pin mill (LV 15 M, Condux-Werk, Hanau, Germany) and subsequently defatted with hexane (Actu-All Chemicals, Oss, The Netherlands) for 24 h using a custom-built Soxhlet extractor. The defatted grits were dried at room temperature under a laminar flow cabinet for an additional 24 h prior to fine milling with an impact mill (Hosokawa Alpine, Augsburg, Germany) operated in the ZPS configuration. Milling was performed at a rotor speed of 8000 rpm and a classifier wheel speed (CWS) of 8000 rpm, with an airflow of 52 m^3^ h^−1^ and a feed rate of 0.4 kg h^−1^. These conditions were previously shown to disrupt the native oat matrix and improve protein enrichment during air classification (Tagle-Freire et al., 2025). The resulting flour was stored in tightly sealed plastic containers at 5 °C until further processing.

Dry fractionation was performed by air classification using an air classifier (ATP, Hosokawa Alpine, Augsburg, Germany) operated at an airflow of 52 m^3^ h^−1^, a feed rate of 0.4 kg h^−1^, and a CWS of 18,000 rpm to maximize protein enrichment (Tagle-Freire et al., 2025). Each dry fractionation run produced a coarse and a fine fraction; only the fine, protein-enriched fraction was retained and is hereafter referred to as oat protein concentrate (OPC).

The conventional AE-IP method was based on the protocol described by Liu (2014), with modifications. As shown in Fig. 1, a slurry was prepared by dispersing defatted oat flour in deionized water (1:5, w/v). The pH was adjusted to 9.5 with 0.5 mol L^−1^ NaOH, and the suspension was mechanically stirred for 1 h at 300 rpm to solubilize proteins, followed by holding at 4 °C for 24 h. After an additional 15 min of stirring, the mixture was centrifuged at 1500 × g for 15 min, and the protein-containing supernatant was collected. Proteins were precipitated by adjusting the supernatant to pH 5.0 with 0.5 mol L^−1^ HCl and standing for 15 min at room temperature. The precipitate was recovered by centrifugation at 13,000 × g for 30 min at 4 °C, resuspended in deionized water (1:3, w/v), and neutralized to pH 7.0 with 0.5 mol L^−1^ NaOH. The resulting slurry was freeze-dried for 72 h using an Epsilon 2-10D LSCplus freeze dryer (Martin Christ Gefriertrocknungsanlagen GmbH, Osterode am Harz, Germany) at a shelf temperature of −20 °C under vacuum (primary drying at ∼1 mbar followed by a final drying step down to 0.01 mbar) to obtain the oat protein isolate produced by AE–IP, hereafter referred to as oat protein isolate (OPI). In addition, the insoluble fraction obtained after alkaline extraction was processed by sequential sieving and washing steps to recover a starch isolate (SI) and a fiber-rich fraction (FC).

The water-only extraction followed the same procedure as AE-IP, except that no pH adjustment was applied during protein solubilization or precipitation (Fig. 1). This approach was based on an aqueous fractionation process previously developed for peas (Möller et al., 2022) and recently applied to oats (Ha et al., 2025). The resulting ingredient was denoted as water-extracted oat protein isolate (OPIW). As in AE-IP, the insoluble pellet was further processed by sequential sieving and washing to recover a starch isolate (SW) and a fiber-rich fraction (FW). All fractionation procedures were performed in triplicate independent batches, and the resulting dried ingredients were stored in sealed plastic containers at 4 °C until analysis.

Mass yield and macronutrient recovery were calculated for all protein ingredients and co-extracted fractions (fiber and starch). Mass yield represents the mass percentage of the ingredient or fraction recovered relative to the initial oat groats. Macronutrient recovery (*Y*_*macronutrient*_) represents the proportion of a given macronutrient (protein, starch, or non-starch carbohydrate; NSC) retained in the ingredient or fraction relative to the initial oat groats and was calculated according to Equation (1)

(1) $$Y_{macronutrient}\left[\%\right]=\frac{\phi_{I}.X_{I}}{\phi_{groats}.X_{groats}}\cdot 100\%$$

where $\phi_{I}$ and $\phi_{groats}$ denote the mass (g) of the ingredient or fraction of interest and the oat groats, respectively, and $X_{I}$ and $X_{groats}$ represent their respective macronutrient contents (% d.b.). Protein recovery was calculated for oat protein ingredients, starch recovery for starch isolates, and NSC recovery for fiber-rich fractions.

### 2.3 Ingredients characterization

#### 2.3.1 Proximate composition

Protein, starch, lipid, and ash contents of the oat ingredients were determined according to AOAC procedures. Protein was quantified by the Dumas combustion method (AOAC 992.23) using a rapid N exceed combustion analyzer (Elementar Analysensysteme, Langenselbold, Germany) and a nitrogen-to-protein conversion factor of 5.83 (Rashed et al., 2024). Starch was determined enzymatically using the Total Starch Assay Kit (Megazyme, Bray, Ireland; AOAC 996.11). Lipid content was measured by Soxhlet extraction with a Soxtherm automated system (C. Gerhardt Analytical Systems, Königswinter, Germany) (AOAC 922.06), and ash by dry incineration at 550 °C for 5 h in a muffle furnace (Carbolite Gero, Hope Valley, UK) (AOAC 923.03). Moisture content was determined gravimetrically with a halogen moisture analyzer (MA37-1, Sartorius Lab Instruments, Gottingen, Germany). Non-starch carbohydrates (NSC), comprising mainly dietary fiber and minor soluble sugars, were estimated by difference as 100 − (protein + starch + lipid + ash), expressed on a dry basis.

All proximate composition analyses were conducted in duplicate (n = 2) using samples obtained by pooling material from independent extraction batches. Pooling was done to ensure sufficient sample mass for all compositional analyses.

#### 2.3.2 Protein profile

Protein profiles of the oat ingredients were analyzed by sodium dodecyl sulfate–polyacrylamide gel electrophoresis (SDS-PAGE) under reducing and non-reducing conditions. Samples were dispersed in water at a protein concentration of 1.6 mg mL^−1^ and mixed 1:1 (v/v) with 2× Laemmli sample buffer with or without dithiothreitol (DTT, 0.1 M). Due to its low solubility, OPIW was dispersed at 3.2 mg mL^−1^ prior to dilution with sample buffer. The mixtures were heated at 100 °C for 10 min prior to loading. Aliquots (20 μL) were loaded onto 12% Mini-PROTEAN precast gels (Bio-Rad Laboratories) together with a pre-stained molecular weight standard (Precision Plus Protein™ Dual Xtra, Bio-Rad). Electrophoresis was performed in Tris–glycine–SDS running buffer at 200 V until protein separation was achieved. Gels were washed in water, stained with Bio-Safe Coomassie G-250, and destained overnight. Gel images were acquired using a GS-900 densitometer (Bio-Rad Laboratories) and used for band visualization.

#### 2.3.3 Ingredient morphology

Ingredient morphology was visualized using scanning electron microscopy (SEM). Powder samples were mounted on 9.5 mm aluminum stubs using carbon adhesive tabs and sputter-coated with gold (Smart Coater, JEOL, Zaventem, Belgium). SEM images were acquired using a JEOL JCM-7000 microscope (JEOL, Zaventem, Belgium) operated at an accelerating voltage of 5 kV. Images were recorded at magnifications of×100, ×300, and ×1000. Representative images were selected and are shown in this manuscript.

#### 2.3.4 Color measurement

Color was measured in the CIELAB color space using a portable dual-beam spectrophotometer (ColorFlex EZ, Hunter Associates Laboratory, Reston, VA, USA). L∗, a∗, and b∗ values were recorded from triplicate measurements (n = 3). Color was determined for oat flour, OPI, and OPIW because alkaline extraction of oat proteins has been associated with darker ingredients due to oxidation and co-extraction of phenolic compounds during processing (Ha et al., 2025).

#### 2.3.5 Ingredient solubility

Ingredient solubility was determined following the protocol of Gaber et al. (2025) with modifications. Briefly, 0.10 g of sample was dispersed in 24.90 g of Milli-Q water (0.4%, w/w). Suspensions were adjusted to pH 7, stirred for 1 h at 500 rpm, and centrifuged at 900 × g for 20 min. An aliquot of the supernatant was collected and dried overnight at 105 °C to determine the dissolved solids content. Ingredient solubility (%) was calculated as the mass of dried solids in the supernatant relative to the initial sample mass. Measurements were performed in triplicate (n = 3).

To evaluate the influence of residual lipids on ingredient solubility and SEM observations, OPIW was analyzed before and after an additional cold defatting step. Defatting was performed by mixing 3 g of sample with 15 mL of hexane and stirring for 1 h at 500 rpm. The suspension was centrifuged at 16,000 × g for 15 min, the supernatant was removed, and the pellet was spread on aluminum foil and air-dried in a fume hood for 1 h prior to solubility measurements and SEM imaging.

### 2.4 Post-heat treatments

Prior to in vitro digestion, each ingredient was suspended in water and subjected to heat treatment at 70 °C and 110 °C, with an unheated sample serving as control (NT). Samples were dispersed in water (1:5, w/w) in sealed 15 mL centrifuge tubes and mixed thoroughly for 5 min. Sealed tubes were used to minimize moisture loss and allow the aqueous dispersions to reach temperatures above 100 °C during heating. Heating at 70 °C was carried out in a temperature-controlled water bath (JULABO TW8, JULABO GmbH, Seelbach, Germany), whereas the 110 °C treatment was applied using a dry heating block (Reacti-Therm III, Thermo Fisher Scientific, Waltham, MA, USA). Equilibration times were experimentally determined using an oat flour dispersion by monitoring the temperature inside the sample tube with a temperature probe (Fig. S1). Based on these measurements, samples were held at the target temperature for 5 min, resulting in total heating times of 8 min (70 °C) and 12 min (110 °C). The thermal treatments were designed as controlled laboratory heat treatments to induce starch gelatinization (70 °C) and starch gelatinization together with oat protein denaturation (110 °C), rather than to directly simulate industrial processing conditions. The extent of these thermal transitions was subsequently evaluated by DSC (section 2.5). After heating, samples were cooled in an ice bath prior to in vitro digestion.

### 2.5 DSC characterization

Oat ingredients were analyzed for thermal transitions associated with protein denaturation and starch gelatinization using differential scanning calorimetry (DSC) (DSC 250, TA Instruments, New Castle, DE, USA). The calorimeter was calibrated for temperature and heat flow using indium. Preliminary measurements performed on dry powders showed no detectable endothermic transitions up to 140 °C; therefore, DSC analyses were conducted using aqueous dispersions. Samples were prepared at a 1:5 (w/w) ratio, and approximately 60 mg was sealed in high-volume pans, with an empty pan used as reference. Measurements were conducted once for each ingredient and treatment condition from 20 to 140 °C at 2 °C min^−1^ to assess protein denaturation. Additional scans at 10 °C min^−1^ were conducted for oat flour and OPC to evaluate starch gelatinization, as these ingredients contained sufficient starch for analysis. Two consecutive heating cycles separated by cooling to 20 °C were conducted at the same heating rate. The absence of peaks during the second cycle confirmed the irreversible nature of the transitions. DSC measurements were conducted for all ingredients before and after thermal treatment at 70 °C and 110 °C (Section 2.4). Data were processed using TRIOS software (TA Instruments). Transition onset temperatures (T_o_), peak temperatures (T_p_), and enthalpies (ΔH) were determined from thermograms plotted as normalized heat flow (W g^−1^) as a function of temperature.

### 2.6 In vitro digestion

Dry oat ingredients were dispersed in water (1:5, w/w) in sealed tubes and subjected to the thermal post-treatments described in Section 2.4. Digestion experiments were performed using equal masses of ingredients rather than normalizing sample weight based on protein content. This approach preserved the intrinsic compositional differences among ingredients, allowing evaluation of the influence of fractionation method on matrix composition and structure. A recombined sample was included (mimic), consisting of OPI blended with the oat starch isolate recovered from the AE-IP process (SI) to match the protein concentration of OPIW. This model ingredient allowed separation of compositional effects from structural differences introduced by the extraction processes.

Static in vitro digestion followed the standardized INFOGEST protocol (Brodkorb et al., 2019) with salivary α-amylase and gastric lipase omitted. Gastric lipase was not required because lipid digestion was beyond the scope of this study. Salivary α-amylase was also omitted, as it was expected to be inactivated early in the gastric phase due to the rapid drop in pH in the static model (Tagle-Freire et al., 2022).

Digestion was conducted in a shaking water bath at 37 °C. The oral phase consisted of mixing the sample dispersion with simulated salivary fluid (SSF) in a 1:1 ratio for 2 min. The gastric phase was performed by addition of simulated gastric fluid (SGF) containing pepsin, adjustment to pH 3.0, and incubation for 2 h. Subsequently, simulated intestinal fluid (SIF) containing pancreatin and bile was added, the pH was adjusted to 7.0, and incubation continued for a further 2 h.

Pancreatin dosing in the intestinal phase was determined from its specific trypsin activity (5.93 ± 0.30 U mg^−1^), whereas pepsin activity (4059 U mg^−1^) was used to achieve the target INFOGEST enzyme activity in the gastric phase. Pancreatin was prepared by suspending the enzyme in SIF, applying ultrasound treatment and centrifugation, and using the supernatant as the enzyme source (Sousa et al., 2023). Sampling was performed at the end of the gastric (2 h) and intestinal (4 h total) phases. Gastric digestion was stopped by increasing pH above 7, and intestinal digestion was terminated by heating at 100 °C for 8 min.

Controls included (i) sample controls prepared with ingredients and simulated digestive fluids but without enzymes or bile, and (ii) enzyme blanks containing enzymes and bile prepared in water at the same concentrations used in the digestion tubes. Sample controls were used to account for background primary amino groups and reducing sugars detected prior to enzymatic hydrolysis rather than being considered as time zero digestibility values, whereas enzyme blanks were used to account for analytical signals originating from digestive enzymes and bile. All digestion experiments and controls were performed in triplicate (n = 3).

### 2.7 Protein digestibility

In vitro protein digestibility after static gastrointestinal digestion was assessed through protein degree of hydrolysis (DH) measurements using the o-phthaldialdehyde (OPA) spectrophotometric assay (Nielsen et al., 2001). The method quantifies the release of primary amino groups derived from free amino acids and peptide termini generated during protein hydrolysis. OPA reacts with primary amines in the presence of thiol reagents to form chromogenic isoindole derivatives, which were quantified spectrophotometrically at 340 nm.

At the end of the gastric and intestinal phases, aliquots were collected immediately after enzyme inactivation (Section 2.6), cooled on ice, and centrifuged (10,000 × g, 30 min) to obtain clear supernatants for OPA analysis. Sample controls were prepared in parallel.

#### **2.7.1** **O-phthaldialdehyde** (**OPA) method**

The OPA reagent was prepared fresh following the procedure described by (Nielsen et al., 2001). Briefly, sodium tetraborate (3.81 g), sodium dodecyl sulfate (0.10 g), and dithiothreitol (0.088 g) were dissolved in Milli-Q water. OPA (0.080 g) was dissolved in ethanol and added to the solution, and the final volume was adjusted to 100 mL. Reagent was protected from light until use.

For each measurement, 20 μL of sample was transferred into a 96-well microplate and mixed with 200 μL OPA reagent using reverse pipetting to avoid bubble formation. The microplate was placed and shaken in a plate reader (MultiSkan FC, Thermo Fisher Scientific, Waltham, USA), and absorbance was measured at 340 nm after a total reaction time of 3 min. Measurements were performed in technical duplicate for each digestion replicate. Quantification was based on a calibration curve prepared from L-serine standards (12.5–150 mg L^−1^). Digesta supernatants were diluted when necessary to fall within the calibration range.

Methanol precipitation of digesta supernatants, as proposed previously (Sousa et al., 2023), was assessed but ultimately not used, as the resulting dilution reduced OPA signals in control samples below the detection limit.

#### 2.7.2 Acid hydrolysis and protein degree of hydrolysis calculation

The total amount of reactive amino groups in each ingredient was determined after complete acid hydrolysis. Samples were hydrolyzed in 6 M HCl at 115 °C for 21 h in sealed tubes, cooled to room temperature, and analyzed by the OPA assay as described before (section 2.7.1).

Protein degree of hydrolysis (DH_P_) was expressed as the proportion of free amino groups released during digestion relative to the total amino groups measured after acid hydrolysis, with correction for both sample controls and enzyme blanks (Equation (2))

(2) $${DH}_{P}\left[\%\right]=\frac{{NH}_{2,digesta}-{NH}_{2,sample\;control}-{NH}_{2,enzyme\;blank}}{{NH}_{2,acid}-{NH}_{2,sample\;control}-{NH}_{2,enzyme\;blank}}\cdot 100\%$$

where ${NH}_{2,digesta}$ represents free amino groups measured in gastric or intestinal digesta, ${NH}_{2,acid}$ represents total amino groups after acid hydrolysis, ${NH}_{2,sample\;control}$ corresponds to samples prepared without digestive enzymes, and ${NH}_{2,enzyme\;blank}$​ represents the OPA signal originating from digestive enzymes in the absence of substrate.

### 2.8 Starch digestibility

In vitro starch digestibility after static gastrointestinal digestion was assessed through starch degree of hydrolysis (DH) measurements using the 3,5-dinitrosalicylic acid (DNS) colorimetric assay (Miller, 1959; Silventoinen-Veijalainen et al., 2024). The assay quantifies reducing sugars released during starch hydrolysis through the reaction of DNS with reducing carbohydrate groups, producing a colored product measured at 540 nm.

At the end of the gastric and intestinal digestion phases, aliquots were collected immediately after enzyme inactivation (Section 2.6), cooled on ice, and centrifuged (4000 × g, 10 min) to obtain clarified supernatants for reducing sugar determination. Digestion controls were prepared in parallel.

#### 2.8.1 Quantification of reducing sugar contents via 3,5-dinitrosalicylic acid (DNS) assay

The DNS reagent was prepared by dissolving 1.0 g 3,5-dinitrosalicylic acid in 30 mL deionized water under heating (∼80 °C), followed by addition of sodium potassium tartrate (30 g) and 20 mL of 2 M NaOH. The volume was adjusted to 100 mL with deionized water, and the reagent was stored protected from light at room temperature until use.

Digesta supernatants (400 μL) were mixed with an equal volume of DNS reagent, vortexed, and heated at 100 °C for 10 min in a water bath. After cooling on ice, 200 μL aliquots were transferred to a 96-well plate, and absorbance was recorded at 540 nm (SpectraMax ABS Plus, Molecular Devices, San Jose, USA). Measurements were performed in technical duplicate for each digestion replicate. Reducing sugars were quantified using a maltose standard curve (0–3.0 mg mL^−1^) and reported as mg maltose equivalents per mL of digesta.

The total reducing sugar potential was determined from complete starch hydrolysis in the samples using the Megazyme Total Starch Assay procedure (method A), with the final reducing sugar concentration quantified in duplicate by the DNS assay to ensure consistency with digesta measurements.

#### 2.8.2 Starch degree of hydrolysis calculation

Starch degree of hydrolysis (DH_S_) was expressed as the proportion of reducing sugars released during digestion relative to the total reducing sugar potential, with correction for both sample controls and enzyme blanks (Equation (3)):

(3) $${DH}_{S}\left[\%\right]=\frac{{RS}_{digesta}-{RS}_{sample\;control}-{RS}_{enzyme\;blank}}{{RS}_{total}-{RS}_{sample\;control}-{RS}_{enzyme\;blank}}\cdot 100\%$$

where ${RS}_{digesta}$ represents reducing sugars measured in gastric or intestinal digesta, ${RS}_{sample\;control}$ corresponds to samples prepared without digestive enzymes, ${RS}_{enzyme\;blank}$ represents the signal originating from digestive enzymes in the absence of substrate, and ${RS}_{total}$ represents the reducing sugar concentration obtained after complete starch hydrolysis.

### 2.9 Statistical analysis

Statistical analyses were performed in R version 4.5.1 (R Core Team, 2024). Data were tested for normality using the Shapiro–Wilk test and for homogeneity of variances using Levene's test. One-way ANOVA was applied to color and solubility measurements. Protein and starch degree of hydrolysis were analyzed using two-way ANOVA with ingredient (7 levels: Flour, OPC, OPI, OPIW, Mimic, C1 and C2) and heat treatment (3 levels: 70 °C, 110 °C, and NT) as fixed factors and their interaction effects, performed separately for the gastric and intestinal digestion phases. When significant effects were detected, Tukey's honestly significant difference (HSD) test was used for post hoc comparisons. For visualization purposes in Fig. 6, ingredient–heat treatment combinations were also compared using one-way ANOVA on the combined factor followed by Tukey's test, with grouping letters used to indicate significant differences. Pearson correlation analysis was used to assess the relationship between solubility and fat content. A p-value <0.05 was considered statistically significant.

## 3 Results and discussion

This section first presents the characterization of the oat ingredients, including composition, protein profile, morphology, and solubility. Thermal transitions related to starch gelatinization and protein denaturation are then presented for the oat ingredients before and after post-heat treatment. Finally, the effects of extraction method and post-heat treatment on in vitro protein and starch digestibility are presented.

### 3.1 Ingredient characterization

**Fig. 2:**
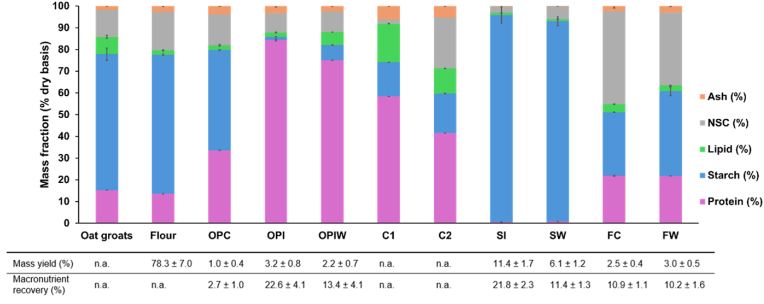
Proximate composition of oat ingredients on a dry matter basis. Bars represent mean values of duplicate analytical measurements of pooled samples (n = 2); error bars indicate the range. The table below the x-axis shows process mass yield (%) and macronutrient recovery (%) calculated from three independent fractionation runs (n = 3). Macronutrient recovery represents the proportion of a given macronutrient retained in the resulting ingredient or fraction relative to the starting material (oat groats). OPC, oat protein concentrate from dry fractionation; OPI, oat protein isolate from AE–IP; OPIW, oat protein isolate from the water-only process; C1 and C2, commercial oat protein concentrates; SI, starch isolate from AE–IP; SW, starch isolate from water-only process; FC, fiber concentrate from AE-IP; FW, fiber concentrate from water-only process; n. a., not applicable; NSC, non-starch carbohydrates (mostly fibers). NSC were calculated by difference as 100 − (protein + starch + lipid + ash).

The proximate composition of the oat ingredients differed markedly depending on the fractionation method (Fig. 2). Dry fractionation produced an oat protein concentrate (OPC) containing 33.6% protein (d.b.) and 46.2% starch (d.b.), reflecting the partial separation of protein- and starch-rich particles during air classification. In contrast, alkaline extraction followed by isoelectric precipitation (AE-IP) yielded the most purified protein ingredient, with OPI containing 84.4% protein (d.b.) and only 1.4% starch (d.b.). The water-only process also generated a protein-rich ingredient, with OPIW containing 75.0% protein (d.b.), although it retained higher levels of starch (7.0% d.b.) and lipids (5.9% d.b.) than OPI.

Detailed compositional data together with moisture content are provided in Table S1. Among the wet extraction methods, AE-IP resulted in higher protein recovery (22.6%) than the water-only process (13.4%) (Fig. 2). Nevertheless, OPIW still exhibited relatively high protein purity (75%), indicating that protein enrichment can occur even without alkaline solubilization. During aqueous extraction, a fraction of oat proteins is dispersed in water, while starch- and fiber-rich materials remain largely insoluble and are removed during centrifugation. Recovery of the protein-containing supernatant therefore results in a protein-enriched fraction, albeit with lower overall protein recovery compared with AE-IP. Similar observations were reported by Ha et al. (2025), who showed that water-based extraction of oats produced protein concentrates with comparable protein content to AE-IP, although at substantially lower protein yield.

The commercial oat protein ingredients also differed considerably from each other (Fig. 2). C1 contained 58.4% protein (d.b.) and 17.7% lipids (d.b.), whereas C2 contained less protein (41.5% d.b.) and substantially more non-starch carbohydrates (23.4% d.b.). These differences indicate that commercial oat protein ingredients can vary widely in composition, likely reflecting differences in industrial processing.

**Fig. 3:**
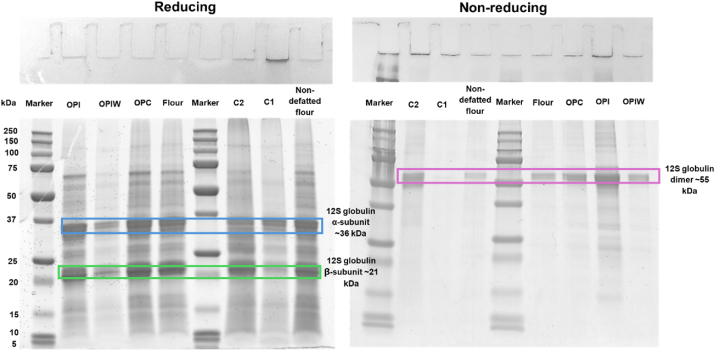
SDS–PAGE of oat ingredients under reducing and non-reducing conditions. Protein samples were prepared at 1.6 mg mL^−1^ in Milli-Q water, except for OPIW (3.2 mg mL^−1^) due to limited solubility. Variations in band intensity are attributed to solubility differences rather than protein concentration. The dark material remaining in the gel wells indicates large protein aggregates or poorly soluble material that did not migrate during electrophoresis. Colored boxes highlight the 12 S globulin dimer (purple) and its subunits (blue:α-subunit; green:β-subunit). OPI, oat protein isolate from alkaline extraction–isoelectric precipitation; OPIW, oat protein isolate from the water-only process; OPC, oat protein concentrate from dry fractionation; C1 and C2, commercial oat protein concentrates.

To determine how fractionation method altered the protein profile of the ingredients, SDS–PAGE analysis was performed under reducing and non-reducing conditions (Fig. 3). The electrophoretic patterns were similar across the oat ingredients, indicating that similar protein profiles were present in all samples independent of the fractionation method. Under reducing conditions, the dominant bands corresponded to the α- and β-subunits of oat 12 S globulins, whereas under non-reducing conditions the band pattern was consistent with the 12 S globulin dimer. These results indicate that the disulfide-linked organization of oat globulins and the overall protein profile were largely preserved regardless of the fractionation method. Similar observations were reported by Ha et al. (2025), who showed that oat proteins obtained through water-based extraction and AE-IP exhibited comparable SDS-PAGE profiles.

Although the overall band patterns were comparable, differences in band intensity were observed among ingredients. Under reducing conditions, OPIW showed weaker migrating bands than the other ingredients. Because SDS–PAGE analysis reflects only the soluble protein fraction obtained during sample preparation, these differences likely arise from variations in protein extractability rather than differences in protein composition. In the non-reducing gels, dark material remained in the loading wells for all ingredients (Fig. 3), indicating that a fraction of the proteins was present as large particles or aggregates unable to migrate into the gel. The amount of this residual material differed between ingredients, suggesting that the extent of aggregation may vary depending on the fractionation method. However, because SDS–PAGE does not allow quantitative evaluation of these aggregates, these observations remain qualitative. Processing steps involved in plant protein fractionation are known to influence protein aggregation state and solubility without necessarily altering the underlying protein composition (Yang et al., 2024).

**Fig. 4:**
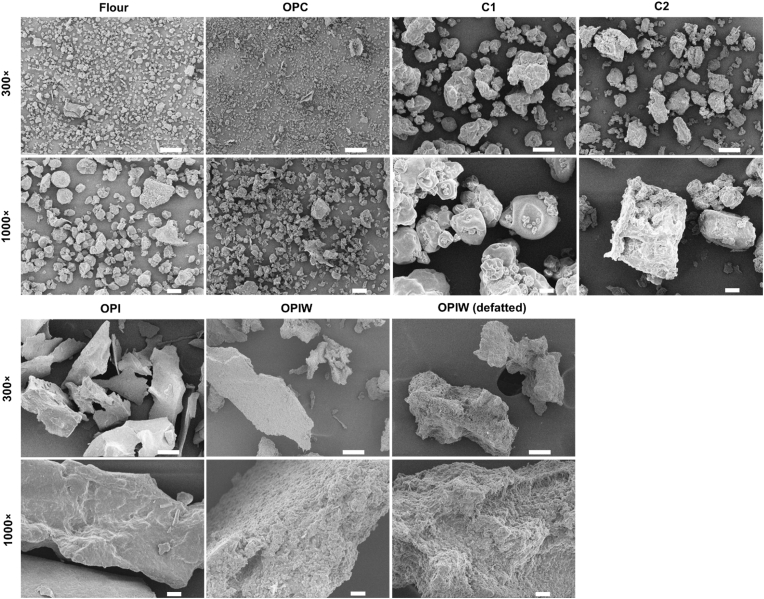
SEM images of oat ingredients at 300× (50 μm scale bar) and 1000× (10 μm scale bar) magnification. OPC, oat protein concentrate from dry fractionation; C1 and C2, commercial oat protein concentrates; OPI, oat protein isolate from alkaline extraction–isoelectric precipitation; OPIW, oat protein isolate from the water-only process; and OPIW (defatted), oat protein isolate from the water-only process after cold defatting.

To further examine whether the type of fractionation influenced the structural organization of the ingredients, particle morphology was analyzed using scanning electron microscopy (SEM). SEM images revealed clear differences in morphology among the oat ingredients (Fig. 4). Flour displayed numerous rounded and oval particles consistent with native starch granules. In contrast, OPC showed much smaller fragmented particles, and the characteristic starch granules visible in flour were no longer distinguishable at the same magnification. This reduction in particle size is consistent with the production of OPC as the fine fraction obtained after air classification of milled oat flour. The commercial oat protein ingredients also exhibited distinct morphologies. C1 consisted mainly of rounded particles that appeared partially hollow or collapsed, whereas C2 displayed more irregular particle shapes with rougher surfaces and a more heterogeneous appearance. These differences likely reflect variations in industrial processing histories.

The wet-extracted ingredients showed different particle morphologies. OPI particles were relatively compact fragments with smooth surfaces, whereas OPIW particles were porous, sponge-like structured particles clearly distinct from those of the other ingredients. Because OPIW retained more lipids than the other ingredients (Fig. 2), SEM images were also obtained after cold defatting to examine whether lipid content contributed to this morphology. Because the sponge-like structure remained similar after lipid removal (Fig. 4), it could be concluded that the observed morphology was not primarily associated with the presence of lipids. Additional SEM images of the starch/fiber-rich fractions obtained during the AE-IP and water-only extraction processes are provided in Fig. S2.

**Fig. 5:**
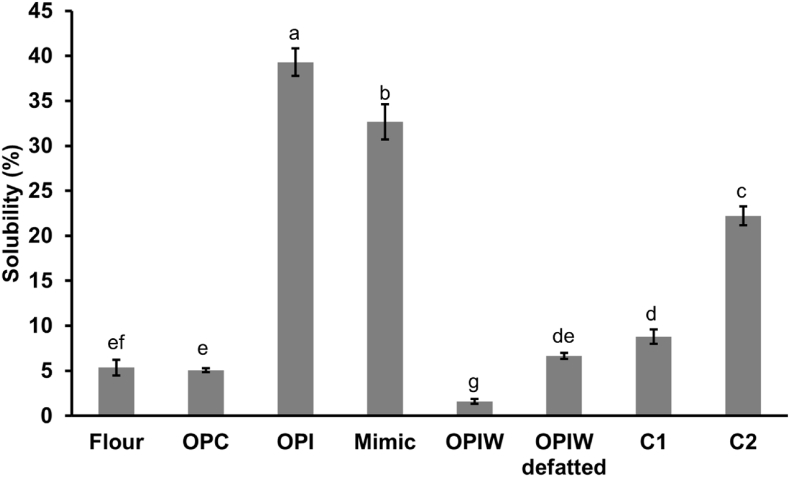
Total solubility (%) of oat ingredients. Bars represent mean ± standard deviation of triplicate measurements (n = 3). Different letters indicate significant differences among samples based on one-way ANOVA followed by Tukey's HSD test (p < 0.05). OPC, oat protein concentrate from dry fractionation; OPI, oat protein isolate from alkaline extraction–isoelectric precipitation; mimic, recombined ingredient formulated to match the protein content of OPIW using OPI; OPIW, oat protein isolate from the water-only process; OPIW defatted, oat protein isolate from the water-only process after cold defatting; C1 and C2, commercial oat protein concentrates.

Solubility differed among the oat ingredients (Fig. 5). OPIW exhibited the lowest solubility, with values approximately 24-fold lower than those of OPI. This limited redispersion behavior was also evident when aqueous dispersions of the ingredients were prepared (Fig. S3). Because OPIW retained more lipids than the other ingredients, lipid content was hypothesized to negatively influence solubility through potential lipid–protein interactions. To examine this, OPIW was cold-defatted, reducing its lipid content to a level comparable to OPI (2.35 vs. 2.00% d.b.). Defatting increased solubility from 1.6% to 6.6%, but the increase was modest relative to the large difference between OPIW and OPI (39.3%), suggesting that residual lipids were not the main factor limiting the solubility of OPIW. This was further verified by Pearson correlation analysis across all ingredients showing no significant relation between lipid content and solubility (r = −0.16, p = 0.71) (Fig. S4).

**Fig. 6:**
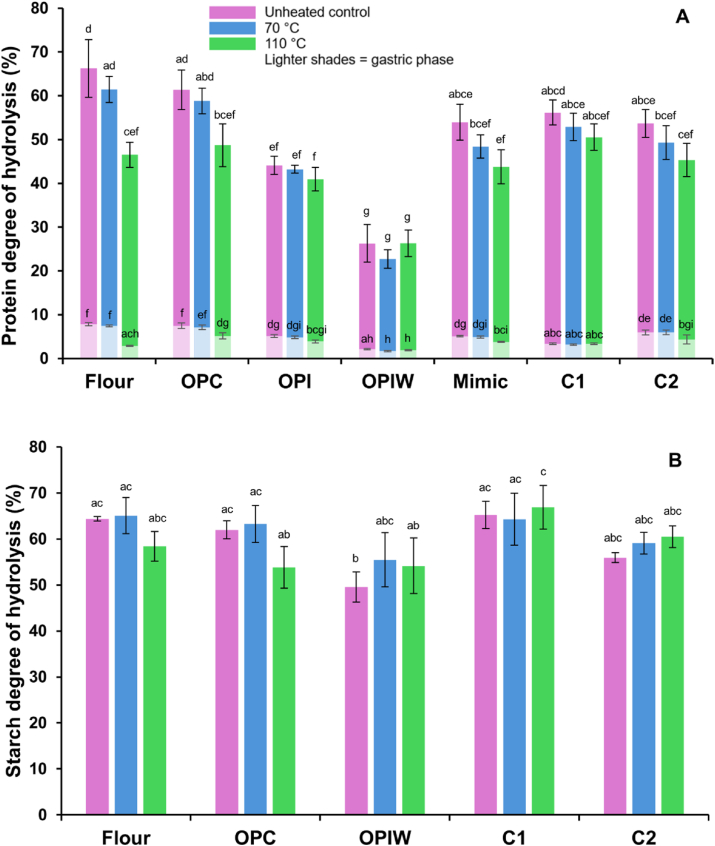
Degree of hydrolysis (DH) of protein (A) and starch (B) after simulated gastric and intestinal digestion of oat ingredients. Bars represent mean ± standard deviation (n = 3). Starch DH after the gastric phase was ∼0 ± 1% and set to 0% for visualization. Starch DH was not determined for OPI due to its very low starch content, and the mimic ingredient was excluded because it was designed for protein digestibility comparison. For display purposes, within each digestion phase, ingredient–heat treatment combinations were compared using one-way ANOVA on the combined factor followed by Tukey's test; different letters above bars indicate significant differences (p < 0.05). OPC, oat protein concentrate from dry fractionation; OPI, oat protein isolate from alkaline extraction–isoelectric precipitation; OPIW, oat protein isolate from the water-only process; mimic, recombined ingredient formulated to match the protein content of OPIW using OPI; C1 and C2, commercial oat protein concentrates.

Protein concentration alone also did not explain the low solubility of OPIW. The mimic ingredient, formulated to match the protein content of OPIW, showed solubility values comparable to OPI rather than OPIW (Fig. 5). Together, these results suggest that structural organization likely played a major role in the limited solubility of OPIW. Supporting this interpretation, SEM images of OPIW before and after cold defatting showed that the sponge-like morphology remained unchanged following lipid removal (Fig. 4; OPIW vs. OPIW-defatted). This morphology may reflect the presence of aggregated protein material that is difficult to redisperse in water, which could contribute to the low solubility observed for this ingredient.

The sponge-like morphology observed for OPIW may be related to the combined effects of the water-only extraction process and subsequent freeze drying. In contrast to alkaline extraction, protein solubilization at near-neutral pH is less likely to induce partial unfolding and aggregation. Conventional alkaline extraction followed by isoelectric precipitation is known to promote conformational changes and the formation of aggregated protein structures in plant protein ingredients (Yang et al., 2024). Proteins recovered through the water-only process may therefore retain a different structural organization prior to drying. During freeze drying, sublimation of ice crystals can generate porous matrices within the dried material (Ratti, 2001), which provides a plausible explanation for the sponge-like particle morphology observed for OPIW. Because both OPI and OPIW were subjected to the same freeze-drying conditions, the distinct particle morphology observed for OPIW is unlikely to originate from the drying process alone. Instead, the results suggest a combined effect in which the extraction step determines the structural organization of the proteins prior to drying, while freeze drying preserves these structural features in the final powder.

Porous structures generated during freeze drying are often associated with improved redispersion due to enhanced water penetration. However, oat proteins are known to exhibit relatively low solubility near neutral pH compared with many pulse proteins, and their functionality is strongly influenced by aggregation state and particle organization (Laursen et al., 2024; McLauchlan et al., 2024). In this context, the sponge-like morphology observed for OPIW particles did not translate into increased solubility, indicating that the structural organization of the protein network formed during extraction and drying may play a more important role than porosity alone. Although the formation mechanism of this structure was not directly investigated in the present study, this interpretation is consistent with the combined SEM and solubility observations.

### 3.2 Starch gelatinization and protein denaturation

In industrial oat processing, kilning is applied to inactivate endogenous lipases and improve storage stability of groats (Li et al., 2025). To evaluate the impact of this treatment on starch and protein thermal state, DSC was performed. Starch gelatinization was assessed in the oat flour, which showed an endothermic peak at ∼68.3 °C (Table 1), consistent with reported oat starch gelatinization temperatures (∼63 °C) (Kaur et al., 2022). The protein denaturation transition in oat flour was weak because of its low protein content (Fig. S5). Therefore, protein denaturation was evaluated in OPC, an ingredient with higher protein content (33.6% d.b.) and no chemical modification relative to oat flour. OPC showed a protein denaturation peak at ∼102.8 °C (Table 1), in agreement with reported oat protein denaturation temperatures (∼110 °C) (McLauchlan et al., 2024). The presence of these thermal transitions suggests that neither starch gelatinization nor protein denaturation occurred during the kilning process. This is consistent with the relatively high denaturation temperature of oat globulins (∼110 °C) (Li et al., 2025) and the limited water availability within the groat matrix, which likely restricted both starch gelatinization and protein denaturation.

**Table 1:** Onset temperature (T_o_), peak temperature (T_p_), and transition enthalpy (ΔH) of thermal transitions in oat ingredients measured by differential scanning calorimetry (DSC) before (NT) and after heat treatments at 70 °C and 110 °C. Flour was analyzed at a heating rate of 10 °C min^−1^ to capture starch gelatinization, while the remaining ingredients were analyzed at 2 °C min^−1^ to assess protein denaturation.

		T_o_ (°C)	T_p_ (°C)	ΔH (J g^−1^)
**Flour**	NT	57.7	68.3	0.6
	70 °C	n.d.	n.d.	n.d.
	110 °C	n.d.	n.d.	n.d.
**OPC**	NT	100.0	102.8	0.3
	70 °C	100.5	107.4	0.4
	110 °C	n.d.	n.d.	n.d.
**OPI**	NT	101.2	109.6	1.4
	70 °C	102.9	110.0	1.3
	110 °C	104.4	111.7	0.4
**OPIW**	NT	102.9	108.9	0.9
	70 °C	102.7	110.2	1.0
	110 °C	106.0	111.8	0.3
**C1**	NT	n.d.	n.d.	n.d.
	70 °C	n.d.	n.d.	n.d.
	110 °C	n.d.	n.d.	n.d.
**C2**	NT	101.2	109.5	0.5
	70 °C	99.6	110.3	0.6
	110 °C	n.d.	n.d.	n.d.

∗OPC, oat protein concentrate from dry fractionation; OPI, oat protein isolate from alkaline extraction–isoelectric precipitation (AE–IP); OPIW, oat protein isolate from the water-only process; C1 and C2, commercial oat protein concentrates; n.d.: not detected.

Post-heat treatment at 70 °C was sufficient to gelatinize starch in Flour and OPC suspensions, whereas treatment at 110 °C resulted in disappearance of the protein transition, indicating complete protein denaturation (Table 1). The wet-extracted ingredients (OPI and OPIW) contained little starch; therefore, their thermograms (Fig. S6) were dominated by protein transitions. Endothermic transitions at ∼108–110 °C were detected in OPI and OPIW without post-heat treatment (NT), suggesting that the wet extraction processes did not induce protein denaturation (Table 1). After heating at 110 °C, residual endothermic transitions remained detectable, indicating incomplete denaturation. The reduced denaturation enthalpy suggests partial alteration of native protein structure during heating, which may also have been influenced by the higher protein content of these isolates compared with Flour and OPC.

Clear differences were observed between the untreated commercial oat proteins (Table 1; C1 and C2). No transition was detected for C1, indicating that proteins were already denatured as a result of its production process. In contrast, C2 exhibited an endothermic transition at 109.6 °C, indicating that proteins were not fully denatured during ingredient production. Heating C2 at 70 °C did not alter the thermogram, whereas heating at 110 °C resulted in near-complete denaturation. Unlike OPI and OPIW, residual transitions were not detected in C2 after heating at 110 °C, which may be related to its lower protein content.

Starch gelatinization transitions were not detectable in most ingredients (Fig. S6), either because of their low starch content (OPI and OPIW) or because starch had likely gelatinized during previous thermal and/or mechanical processing steps (C1 and C2).

### 3.3 Effect of the extraction method on protein and starch in vitro digestion

Protein digestibility was significantly affected by ingredient, post-heat treatment, and their interaction in both digestion phases according to the two-way ANOVA (p < 0.01). To isolate the influence of fractionation method from post-extraction heat treatments on digestibility, the non-heat-treated ingredients (NT) were first compared (Fig. 6). Flour (66.2%) and OPC (61.4%) showed the highest degree of protein hydrolysis, whereas OPI exhibited lower protein hydrolysis (44.1%) and OPIW showed the lowest DH after gastric and intestinal digestion (26.3%) (Fig. 6A).

The higher protein DH observed for Flour and OPC compared with the wet-extracted ingredients indicates that protein isolation did not increase digestibility in the oat ingredients studied here. One possible explanation is that the starting flour used for all fractionation routes had already undergone extensive fine milling, which likely disrupted the native oat matrix and increased protein accessibility during digestion. Despite the presence of starch and other non-protein components in Flour and OPC that could potentially hinder proteolysis, relatively high protein hydrolysis was observed. Similar observations have been reported previously for dry and wet fractionated protein ingredients, where differences in digestibility could not be explained by composition alone, highlighting the importance of matrix organization and processing history (Opazo-Navarrete et al., 2019).

**Fig. 7:**
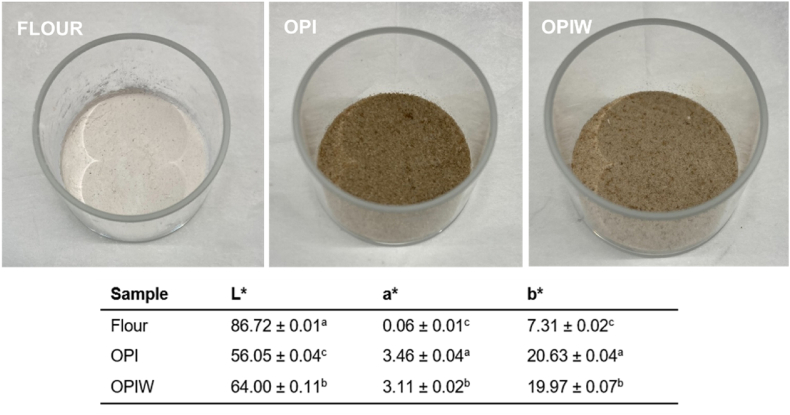
Images of oat flour, OPI, and OPIW, and their corresponding color parameters. Color was expressed in the CIELAB color space (L∗, lightness; a∗, green–red axis; b∗, blue–yellow axis). Values represent mean ± standard deviation (n = 3). Different letters within each parameter indicate significant differences among samples (one-way ANOVA followed by Tukey's test, p < 0.05). OPI, oat protein isolate from alkaline extraction–isoelectric precipitation; OPIW, oat protein isolate from the water-only process.

Previous work has shown that alkaline extraction of oat proteins can promote oxidation and co-extraction of phenolic compounds, resulting in darker oat protein ingredients (Ha et al., 2025). Consistent with these observations, OPI exhibited a darker appearance than Flour and OPIW (Fig. 7), suggesting that similar processing-related changes may have occurred during AE-IP. Phenolic compounds are known to interact with proteins and digestive enzymes and may influence proteolytic activity (Ozdal et al., 2013). However, because phenolic content and protein–phenolic interactions were not measured directly in the present study, the contribution of these effects remains uncertain.

Clear differences were also observed between OPI and OPIW. OPIW consistently showed the lowest protein DH, although SDS–PAGE profiles were broadly comparable and DSC indicated no evidence of extensive protein denaturation, suggesting that differences in protein profile or major denaturation were unlikely to explain its lower digestibility. Instead, the results point toward differences in ingredient morphology and redispersion behavior. As discussed in Section 3.1, OPIW showed the lowest solubility, weaker migrating bands under reducing SDS–PAGE conditions, and a sponge-like morphology that persisted after cold defatting, all of which point to poor redispersion behavior and limited protein accessibility during digestion. In addition, the mimic ingredient, formulated with OPI to match the protein content of OPIW, showed substantially higher DH, indicating that protein concentration alone did not explain the behavior of OPIW. Oat proteins are known to have limited aqueous solubility near neutral pH, and their functionality depends strongly on colloidal organization and self-association in aqueous systems (McLauchlan et al., 2024; Wouters and Nicolai, 2024). Therefore, the lower protein DH of OPIW is more likely related to the formation of poorly redispersible aggregates during water-only extraction and freeze drying, which may have restricted enzyme access during digestion. In contrast, the higher solubility of OPI likely promoted better dispersion and enzyme accessibility during digestion.

Commercial oat ingredients C1 and C2 differed in composition, morphology, solubility, and thermal behavior, yet no significant differences in protein DH were observed between them (p = 0.99) (Fig. 6A). For example, C1 had higher protein and lipid contents and lower solubility, whereas C2 contained more non-starch carbohydrates and showed higher solubility. In addition, DSC analysis indicated the absence of a detectable protein denaturation transition in C1, while a clear transition was observed in C2. These observations indicate that protein digestibility in oat ingredients cannot be predicted from ingredient solubility or protein thermal state alone and likely depends on multiple processing-related factors. However, the unknown processing history of these samples limits further interpretation.

In addition to protein digestibility, starch digestibility was also evaluated (Fig. 6B). For intestinal starch DH, two-way ANOVA showed a significant effect of ingredient and a significant interaction between ingredient and heat treatment (p < 0.05), whereas heat treatment alone did not significantly affect starch DH (p = 0.16). OPIW was the only ingredient with significantly lower starch DH (p < 0.05), whereas the remaining ingredients did not differ from each other. Gastric-phase starch DH values were close to zero, consistent with the absence of α-amylase in the oral phase of the digestion protocol. The reduced starch DH observed for OPIW suggests lower accessibility of residual starch to digestive enzymes. This is consistent with the physicochemical characteristics previously reported for this ingredient, including its low solubility and sponge-like aggregated morphology (Section 3.1), which may limit dispersion and restrict enzyme access during digestion. Although the fractionation route appeared to influence starch digestibility, this effect was largely driven by the behavior of OPIW and should therefore be interpreted with caution.

### 3.4 Effect of heat treatment on protein and starch in vitro digestion

Post-heat treatment influenced protein DH, and the effect varied among ingredients, consistent with the significant interaction between ingredient and heat treatment observed in the two-way ANOVA (p < 0.05). Significant changes were observed mainly in Flour and OPC. Heating at 110 °C reduced protein DH in these ingredients during both the gastric and intestinal phases, whereas no significant differences were observed for the remaining samples. When the temperature was limited to 70 °C, protein DH remained unchanged (p > 0.05). The absence of changes at 70 °C is notable considering that DSC analysis (Section 3.2) indicated complete starch gelatinization in Flour and OPC at this temperature. Despite this structural change, protein DH remained unaffected, suggesting that starch gelatinization alone did not substantially modify enzyme accessibility to proteins under the applied digestion conditions.

The stronger reduction in protein DH observed after heating at 110 °C in Flour and OPC is consistent with the DSC results (Section 3.2), which showed disappearance of the protein denaturation peak following this treatment. In contrast, the remaining ingredients produced in this study retained a residual denaturation peak, indicating a less pronounced thermal transition. This suggests that the lower protein DH in Flour and OPC was associated with a greater protein structural modification during heating. In oat systems, such thermal modification may promote protein association or aggregation following denaturation (Zhao et al., 2004), potentially reducing enzyme accessibility and limiting proteolysis during digestion. In addition, Flour and OPC contained higher levels of non-starch carbohydrates (mainly fibers, including β-glucans) (Fig. 2), which may further influence enzyme accessibility by increasing the viscosity of the dispersed system and limiting enzyme–substrate interactions during digestion (Wood, 2007). The images presented in Fig. S3 illustrate the changes in suspension consistency observed after dispersion in water and thermal treatment, ranging from liquid suspensions to thicker slurries or pastes depending on the ingredient. Such differences in dispersion behavior may also have influenced enzyme accessibility during digestion.

The effect of post-heat treatment on starch hydrolysis was also examined. In contrast to the protein DH results, starch DH showed no significant differences after heating at 70 °C or 110 °C (p > 0.05). This was unexpected because DSC indicated starch gelatinization in Flour and OPC (Section 3.2), which typically increases susceptibility to amylolysis by disrupting crystalline order and promoting granule swelling (Kim and White, 2012; Wang and Copeland, 2013).

Several factors may help explain these results. First, all oat ingredients were derived from finely milled oat flour, which already contains disrupted cellular structures and small particle sizes. Under such conditions, starch may already be readily accessible to digestive enzymes, reducing the impact of additional structural changes induced by heating. Second, starch hydrolysis was assessed only at the end of the intestinal phase. If heat treatment primarily affects the rate rather than the final extent of digestion, potential differences in digestion kinetics may no longer be detectable at this endpoint. Moreover, the digestion model and analytical approach used to evaluate starch hydrolysis may influence the observed outcomes, as different in vitro protocols capture distinct aspects of the digestive process (Freitas et al., 2025). Finally, although thermal processing can increase starch susceptibility to amylolysis, the presence of other oat components such as proteins, lipids, and non-starch polysaccharides, including β-glucan may still influence enzyme accessibility within the complex flour matrix (Björck et al., 1994).

### 3.5 Processing history as a determinant of oat protein ingredient digestibility

The results of this study indicate that the digestive behavior of oat protein ingredients cannot be explained solely by differences in composition generated during fractionation. Instead, the findings suggest that processing also influences digestibility through ingredient solubility and particle morphology. These properties may contribute to differences in protein digestibility by affecting the accessibility of proteins to digestive enzymes.

The effect of post-heat treatment on protein digestibility outcomes further supports this interpretation. The same heat treatments produced different effects depending on the ingredient, indicating that the impact of thermal processing depended not only on temperature but also on the ingredient matrix resulting from fractionation. Heating at 70 °C induced starch gelatinization in Flour and OPC without affecting protein DH, whereas heating at 110 °C reduced protein DH mainly in these ingredients. Flour and OPC also showed disappearance of the protein denaturation peak after heating at 110 °C, suggesting that denaturation and possible aggregation of oat proteins contributed to the reduction in protein digestibility. In contrast, the wet-fractionated oat protein ingredients (OPI and OPIW) retained a residual denaturation peak after heating. One possible explanation is that Flour and OPC contained lower protein concentrations than the more refined oat protein ingredients, which may have influenced the thermal behavior of the system. However, the different responses may also reflect physicochemical changes associated with fractionation and subsequent processing that were not directly evaluated in this study.

In contrast to protein digestibility, starch digestibility appeared less sensitive to the applied heat treatments. One possible explanation is that the finely milled oat flour used as starting material already contained disrupted grain structures that increased starch susceptibility to enzymatic hydrolysis. Under such conditions, additional structural changes induced by fractionation or heating may have had limited influence on the final extent of starch hydrolysis.

In food applications, thermal processing conditions are generally selected based on technological requirements and food safety considerations rather than digestibility alone. However, the present results indicate that oat ingredients produced through different fractionation routes may respond differently to the same thermal treatment. Therefore, ingredient selection may influence not only the techno-functional properties of the final product, but also the extent to which protein digestibility is affected during processing. This may be particularly relevant for oat-based products subjected to thermal treatments after ingredient production, such as high-protein beverages, yogurt alternatives, and meat analogue formulations, where heating may contribute to thickening, gel formation, and broader product structuring. In these applications, heat-induced denaturation and aggregation may contribute to desirable techno-functional properties, but may also influence protein digestibility depending on the ingredient used (Brückner-Gühmann et al., 2021; McLauchlan et al., 2024).

## 4 Conclusions

This study evaluated how fractionation method and post-heat treatment influence the in vitro digestibility of oat protein ingredients containing both protein and starch. The results show that processing history plays a key role in shaping protein digestibility. Different fractionation methods produced ingredients with distinct compositions and particle morphologies, which were associated with substantial differences in protein digestibility. In particular, the low digestibility of the water-only extracted ingredient (OPIW) could not be attributed to a single measured factor but was associated with its low solubility and sponge-like particle morphology. The response to post-heat treatment differed among ingredients. Heating at 70 °C did not affect protein digestibility, whereas heating at 110 °C reduced protein digestibility in flour and OPC, consistent with the loss of protein denaturation transitions observed by DSC. In contrast, starch digestibility showed limited variation among ingredients and was unaffected by post-heat treatment.

Overall, these findings demonstrate that differences in digestibility among oat protein ingredients cannot be explained by composition alone and are linked to the processing history of the ingredients. Consideration of both fractionation method and post-heat treatment may therefore support the development of oat protein ingredients with targeted nutritional properties. Establishing how these processing-induced differences influence nutrient absorption and postprandial responses represents an important next step toward understanding the nutritional consequences of oat processing.

## Consent for publication

The manuscript has been approved for publication by all authors.

## Ethical approval

Not applicable.

## CRediT authorship contribution statement

Danny Tagle-Freire: Conceptualization, Methodology, Validation, Formal analysis, Investigation, Writing – Original Draft, Visualization. Jesse J. Kuiper: Investigation, Visualization. Michiel Ohms: Investigation, Visualization. Yuanfu Chiang: Investigation, Visualization. Markus Stieger: Conceptualization, Writing – Review and Editing, Supervision, Funding acquisition. Ciaran Forde: Conceptualization, Writing – Review and Editing, Supervision, Project administration, Funding acquisition. Maarten Schutyser: Conceptualization, Writing – Review and Editing, Supervision, Funding acquisition.

## Declaration of generative AI and AI-assisted technologies in the manuscript preparation process

During the preparation of this work the authors used ChatGPT in order to improve readability and language of the work. After using this tool, the authors reviewed and edited the content as needed and take full responsibility for the content of the published article.

## Funding

This work is part of the “Metabolic Impact of Future Food Processing – Meta Pro” project (LWV22098), funded by the public-private consortium TKI (10.13039/501100020068Topconsortium voor Kennis en Innovatie) in the Netherlands. The funding consortium had no role in the design of the study, data collection, analysis or interpretation, writing of the report, or the decision to submit the article for publication.

## Declaration of competing interest

The authors declare that they have no known competing financial interests or personal relationships that could have appeared to influence the work reported in this paper.

## Data Availability

YODAData underlying the research on "Processing history shapes in vitro digestibility of protein and starch within oat protein ingredients: Influence of fractionation and thermal treatment." (Original data)
