# Supplementary material for: Fractionation methods and post-heat treatment shape in vitro protein and starch digestibility of oat protein ingredients
Source: Curr Res Food Sci. 2026 Jun 15;13:101477. doi: 10.1016/j.crfs.2026.101477 (PMC13292547; doi:10.1016/j.crfs.2026.101477)
Supplement: Multimedia component 1 [file mmc1.docx]

**Supplementary material for the paper “Fractionation methods and post-heat treatment shape in vitro protein and starch digestibility of oat protein ingredients”**

**
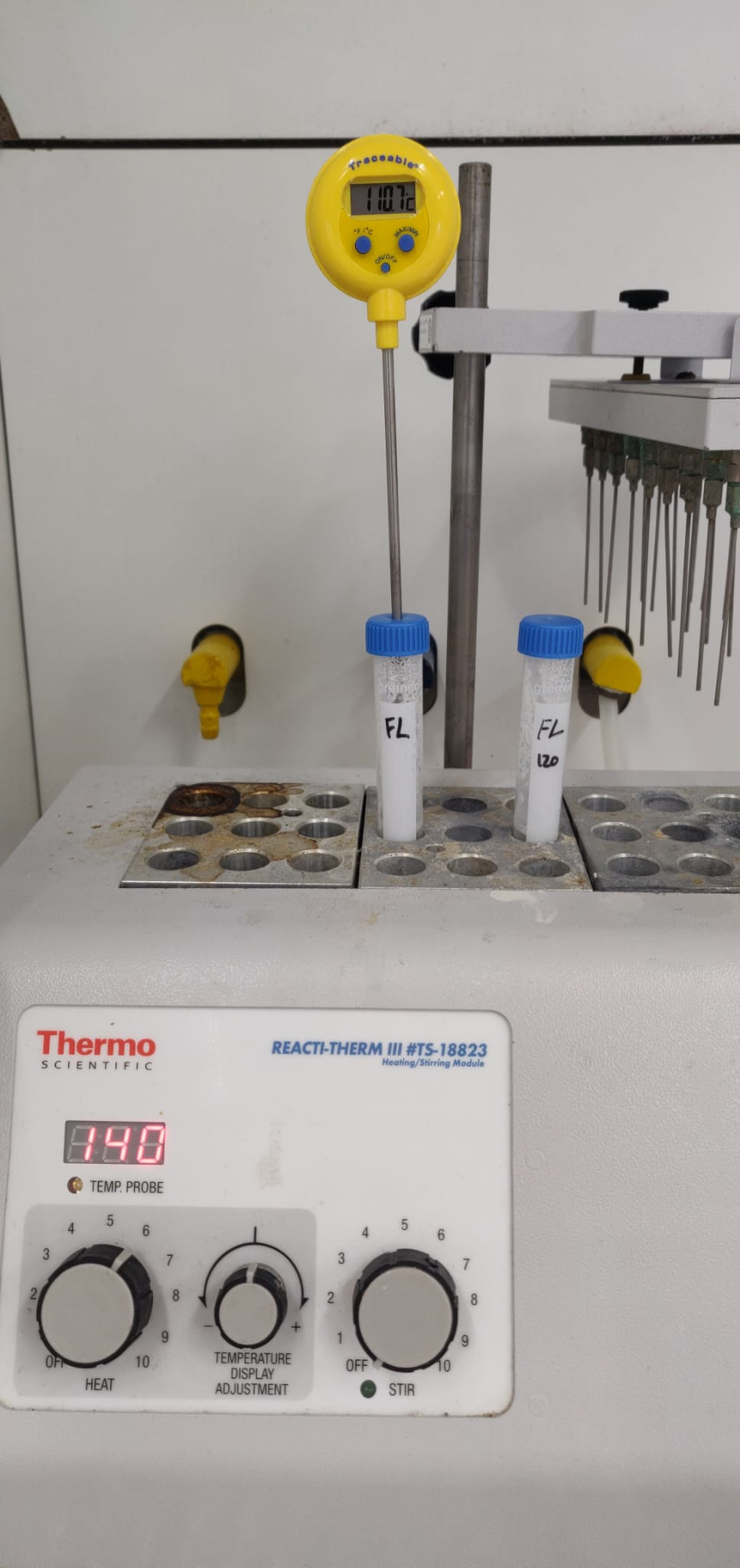
**

**Fig. S1.** Experimental setup used to determine equilibration times for the thermal treatments. A temperature probe was inserted into an oat flour dispersion inside a sealed centrifuge tube to monitor the internal sample temperature during heating.

**Table S1.** Proximate composition, mass yield, and macronutrient (protein, starch, and non-starch carbohydrates; NSC) recoveries of oat ingredients. NSC were calculated by difference as 100 – (protein + starch + lipid + ash). Macronutrient recoveries represent the proportion of a given macronutrient retained in the ingredient or fraction relative to the starting material (oat groats). Average compositional values are based on duplicate analytical measurements of pooled samples (n = 2). Mass yield and macronutrient recoveries were calculated from independent fractionation runs (n = 3). All values are reported on a dry basis.

|  | Moisture (%) | Protein (%) | Starch (%) | Lipid (%) | NSC (%) | Ash (%) | Mass yield (%) | Macronutrient recovery (%) |
| --- | --- | --- | --- | --- | --- | --- | --- | --- |
| Oat groats | 10.9 | 14.2 | 58.4 | 7.5 | 18.2 | 1.7 | - | - |
| Flour | 10.7 | 13.6 | 64.0 | 2.0 | 17.6 | 2.8 | 78.3 | - |
| OPC | 7.3 | 33.6 | 46.2 | 2.2 | 14.3 | 3.8 | 1.0 | 2.7 |
| OPI | 2.8 | 84.4 | 1.4 | 2.0 | 8.8 | 3.4 | 3.2 | 22.6 |
| OPIW | 2.5 | 75.0 | 7.0 | 5.9 | 9.4 | 2.6 | 2.2 | 13.4 |
| C1 | 4.4 | 58.4 | 15.7 | 17.7 | 2.0 | 6.2 | - | - |
| C2 | 3.4 | 41.5 | 18.2 | 11.5 | 23.4 | 5.3 | - | - |
| SI | 12.1 | 0.7 | 95.2 | 1.0 | 2.9 | 0.2 | 11.4 | 21.8 |
| SW | 7.0 | 0.8 | 92.2 | 0.9 | 5.8 | 0.2 | 6.1 | 11.4 |
| FC | 12.5 | 21.9 | 29.2 | 3.7 | 42.8 | 2.4 | 2.5 | 10.9 |
| FW | 4.2 | 21.8 | 39.0 | 2.6 | 33.6 | 3.0 | 3.0 | 10.2 |

*NSC: non-starch carbohydrates (mostly fibers); OPC: oat protein concentrate from dry fractionation; OPI: oat protein isolate from AE-IP; OPIW: oat protein isolate from water-only process; AE-IP: alkaline extraction – isoelectric precipitation; C1 and C2: commercial oat protein concentrates; SI: starch isolate from AE-IP; SW: starch isolate from water-only process; FC: fiber concentrate from AE-IP; FW: fiber concentrate from water-only process; ‘-‘: not applicable.


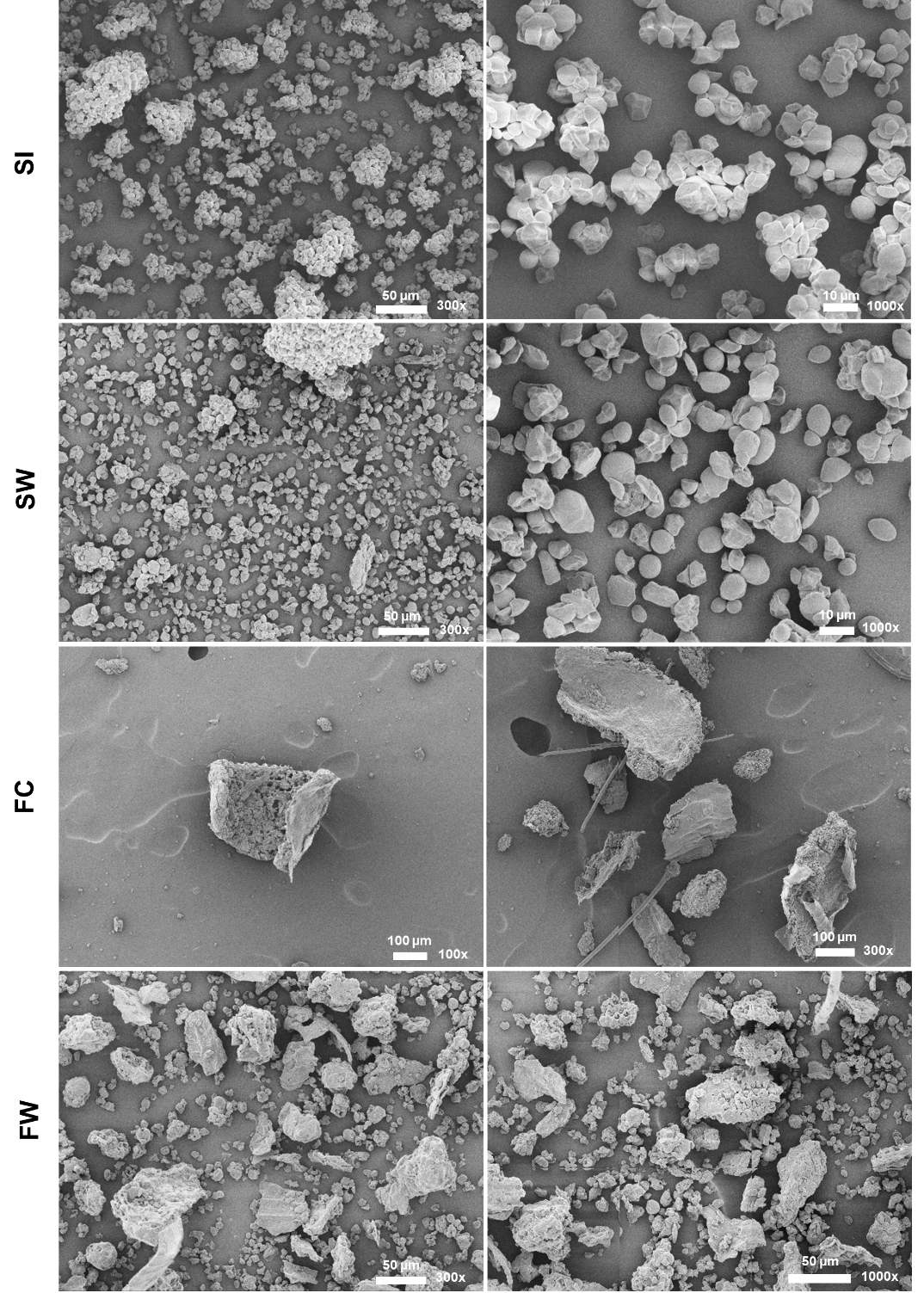


**Fig. S2.** SEM images of starch isolate from AE-IEP (SI), starch isolate from water-only process (SW), fiber concentrate from AE-IEP (FC), and fiber concentrate from water-only process (FW). Magnifications are indicated alongside the scale bars.

**
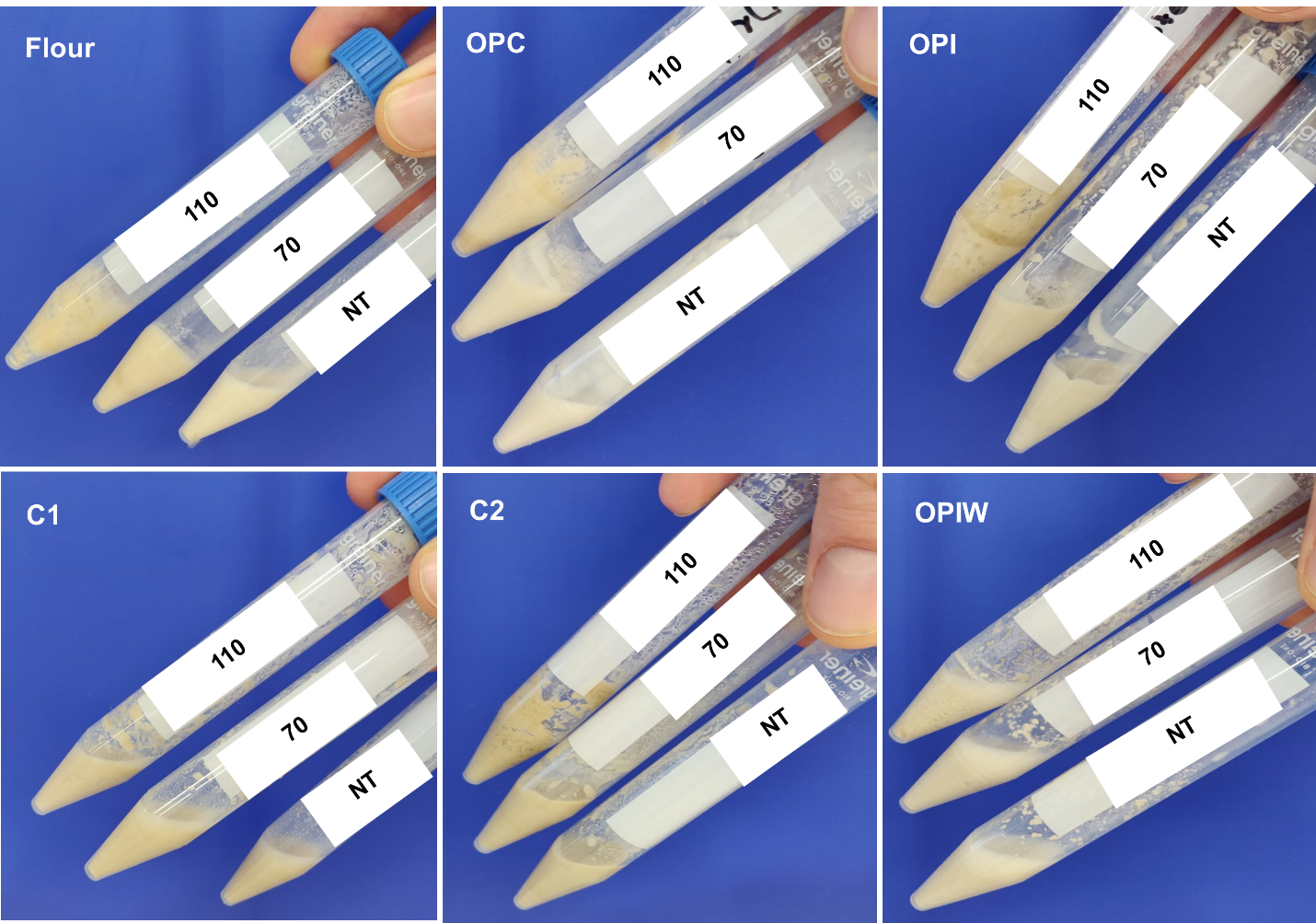
**

**Fig. S3**. Images of oat ingredient dispersions in water (1:5, w/v) prepared without heat treatment (NT) and after heating at 70 °C (70) or 110 °C (110). OPC, oat protein concentrate from dry fractionation; OPI, oat protein isolate from Alkaline extraction-Isoelectric precipitation (AE-IP); OPIW, oat protein isolate from the water-only process; C1 and C2, commercial oat protein concentrates.


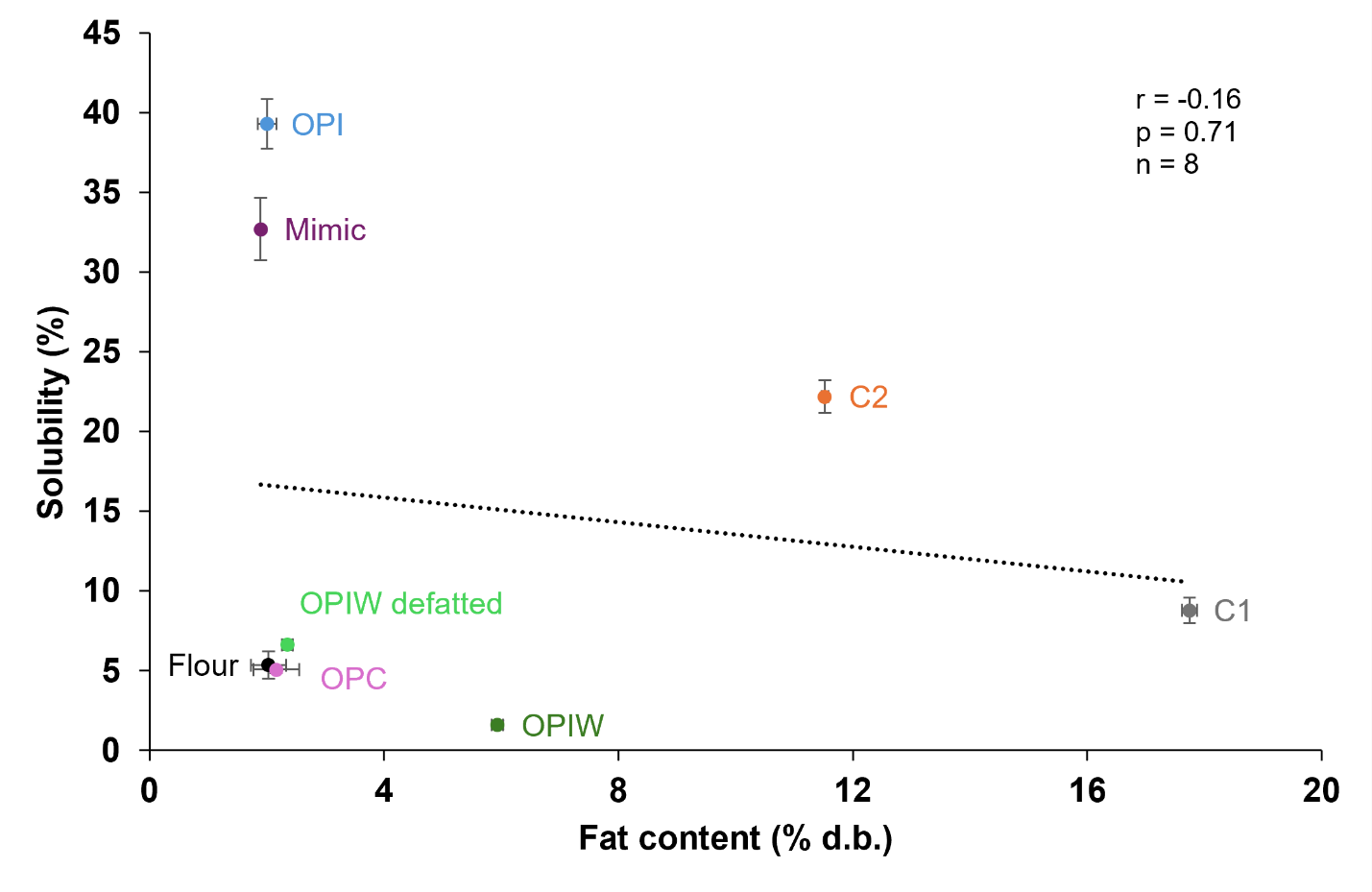


**Fig. S4.** Relationship between solubility (%) and fat content (% d.b.) of oat ingredients. Points represent mean values of analytical triplicates (n = 3), with error bars indicating standard deviation. Each ingredient is represented by a distinct color. The linear regression line is shown. The Pearson correlation coefficient (r), corresponding p-value, and number of observations (n) are indicated in the plot. OPC, oat protein concentrate from dry fractionation; OPI, oat protein isolate from alkaline extraction–isoelectric precipitation (AE-IP); OPIW, oat protein isolate from water-only extraction; mimic, model ingredient formulated to match the protein content of OPC using OPI; C1 and C2, commercial oat protein concentrates.


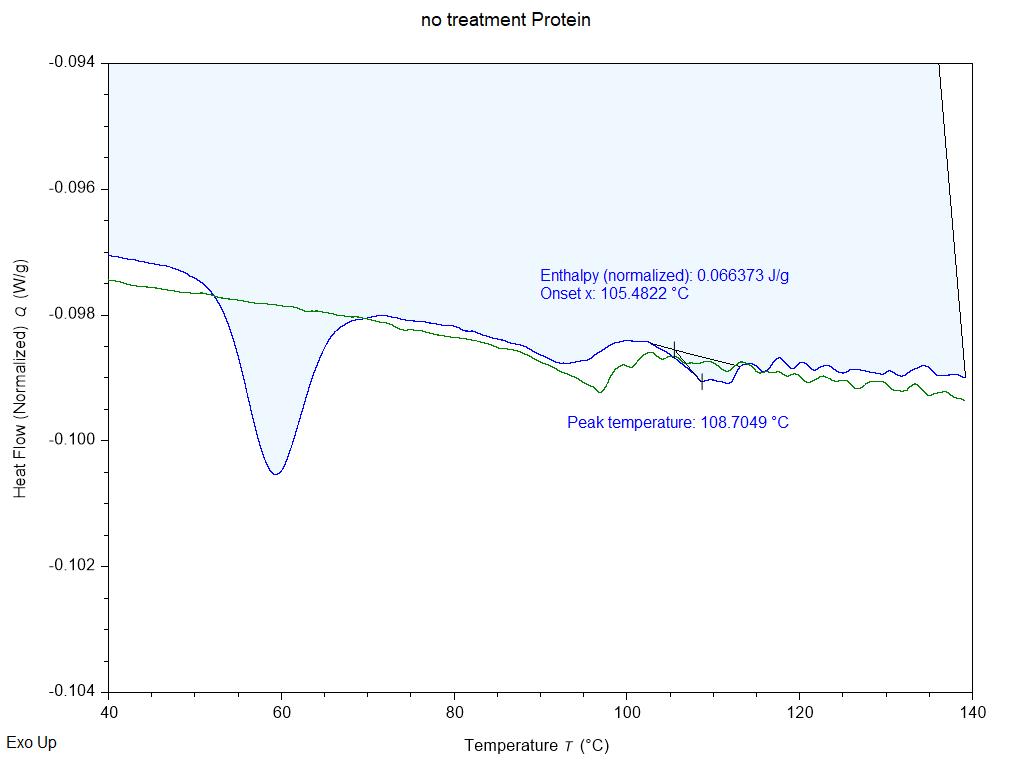


**Fig. S5.** Differential scanning calorimetry (DSC) thermogram of oat flour without heat treatment (NT) recorded at a heating rate of 2 °C/min up to 140 °C. The enthalpy, onset, and peak temperatures of the endothermic transition attributed to protein denaturation are indicated. The weak peak is likely due to the low protein content of Flour (~14% d.b.). Conclusions regarding protein denaturation were drawn from the oat protein concentrate obtained via dry fractionation (Table 1).


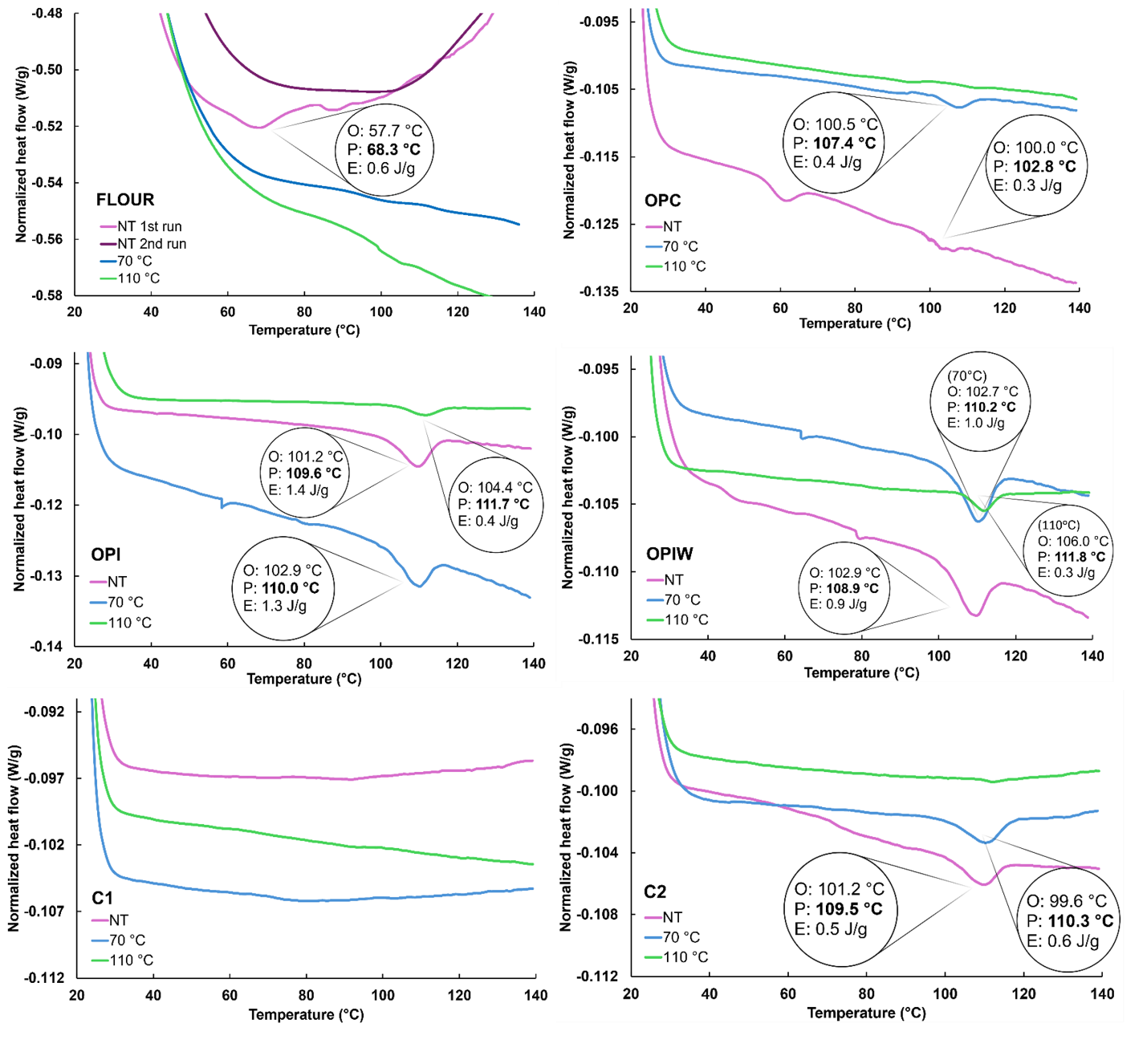
**Fig. S6.** Differential scanning calorimetry (DSC) thermograms of oat ingredients before (NT) and after heat treatments at 70 °C and 110 °C. Flour was analyzed at a heating rate of 10 °C min⁻¹ to capture starch gelatinization, while the remaining ingredients were analyzed at 2 °C min⁻¹ to assess protein denaturation. Onset temperature (O), peak temperature (P), and transition enthalpy (E) are indicated for each endothermic event. To verify the presence of endothermic transitions, two consecutive runs were conducted at the same heating rate. The absence of peaks in the second run confirmed the irreversible nature of the thermal transitions. As an example, two consecutive DSC scans of untreated oat flour (NT) are shown, while only the first run is presented for the remaining samples. OPC, oat protein concentrate from dry fractionation; OPI, oat protein isolate from Alkaline extraction-Isoelectric precipitation (AE-IP); OPIW, oat protein isolate from the water-only process; C1 and C2, commercial oat protein concentrates.
